# Physical activity reduces internet addiction among “post-00” college students: The mediating role of coping styles

**DOI:** 10.3389/fpsyg.2022.1052510

**Published:** 2023-02-08

**Authors:** Meichao Cheng, Shun Wang, Ya Wang, Rong Zhang, Likai Qin

**Affiliations:** ^1^School of Physical Education, Shandong University, Jinan, China; ^2^School of Physical Education, Huaibei Normal University, Huaibei, China; ^3^School of Life Sports, Namseoul University, Cheonan, Republic of Korea

**Keywords:** physical activity, internet addiction, mediating role, “post-00” college students, coping styles

## Abstract

**Objective:**

To explore the role of coping styles (CS) in mediating the relationship between physical activity (PA) and internet addiction (IA), and to provide a theoretical basis for the prevention and treatment of IA among Chinese “post-00” college students.

**Methods:**

A total of 410 university students from five universities in Anhui Province were surveyed using the Chinese Internet addiction scale, the Coping Styles Scale for Chinese University Students, and the International Physical Activity Scale Short Form.

**Results:**

(1) Boys are better than girls in PA. However, there was no significant difference between male and female students on CS and IA. (2) PA was positively correlated with CS (*r* = 0.278, *p* < 0.01), PA was negatively correlated with IA (*r* = −0.236, *p* < 0.01), CS was negatively correlated with IA (*r* = −0.560, *p* < 0.01). (3) PA was negatively predicted IA (*B* = −0.198, *p* < 0.01), PA was positively predicted CS (*B* = 0.986, *p* < 0.01), CS was negatively predicted IA (*B* = −0.065, *p* < 0.01). (4) CS plays a partially mediating role between PA and IA, with a mediating effect proportion of 48.33%.

**Conclusion:**

PA can improve IA not only directly for university students, but also indirectly by increasing CS. The intervention for IA of “post-00” college students can start by increasing PA and improving CS.

## Introduction

With its unique communication advantages, the Internet has become the most popular information dissemination platform and is gradually affecting all aspects of people’s lives. While the Internet has brought convenience to people, it has also produced negative effects that cannot be ignored. Among them, internet addiction (IA) has attracted the attention of all sectors of society. By December 2021, the number of Chinese Internet users had reached 1.032 billion, and the proportion of Internet users using mobile phones to access the Internet was as high as 99.7% [China National Network of Industries (CNNIC), 2022]. The International Classification of Diseases, Eleventh Revision (ICD-11), officially classifies “Gaming disorder” as a disorder (ICD-11, 2019). IA is a pathological form of Internet use or deviant behavior, which refers to a chronic or cyclical state of obsession caused by repeated use of the Internet, accompanied by psychological symptoms of addiction such as increased tolerance and withdrawal reactions (Spada, 2014). IA has a particularly negative impact on university students, such as affecting sleep quality, limiting leisure activities, lowering school grades, increasing absenteeism, increasing psychological and social stress, increasing loneliness and depression, and even increasing suicide thoughts (Ding et al., 2011, 2018; Wei et al., 2020; Ye et al., 2021; Du and Zhang, 2022; Lu et al., 2022; Zewde et al., 2022).

The “post-00” university students are those born between January 1, 2000 and December 31, 2009, and a high percentage of them are students by profession, mainly only children (Ma, 2019). They have the following characteristics: growing up in China’s mobile network era, they are more dependent on the Internet; they have a higher standard of living, but they are less able to control themselves; they are under greater academic and psychological pressure, and some of them lack interest in learning or are even bored with school (Lv, 2019; Ho, 2021). 2018, the “post-00s” college students officially entered the university campus, and after entering the university, they face an unfamiliar environment and a change in their lifestyle. In 2020, after the explosion of COVID-19, almost all university students were isolated at home in order to stop the spread of the epidemic. The internet has become the most important tool for communication, entertainment and learning for university students living at home (Chang and Hou, 2022). The study found that IA was more likely to occur during COVID-19 due to increased network use (Nagaur, 2020). In addition, the fear and uncertainty of the epidemic has led to more severe depression, anxiety, and fear among university students, causing them to need to seek safety from cyberspace, resulting in IA (Shader, 2020). In order to overcome the negative effects of IA, scholars have proposed different countermeasures. The three main methods of treatment regarding IA are psychological intervention, medical and pharmaceutical interventions. University students are more resistant to psychological treatment and often give up treatment because of the limitations of pharmaceutical interventions, such as long lead times, high costs and side effects. Physical activity (PA) has advantages in IA treatment as a low-cost and easily available tool.

## Theories and hypotheses

By analyzing the causes and dangers of IA, we found that the mechanisms of PA influence on IA are very complex, including personality traits, biological factors, environmental factors and other factors. Theories that combine these factors to explain IA include the ACE model, the Cognitive-Behavioral model and the Theories of Usage and Gratification. Davis proposed the Cognitive-Behavioral Model, which divides the influences of IA into distal necessary and proximal sufficient conditions, with distal necessary conditions including vulnerability and life events, and proximal sufficient conditions including non-adaptive cognition, social isolation and lack of social support (Davis et al., 2011). Parker thinks that when individuals use the Internet, they will get different levels of satisfaction according to their psychological needs, such as socializing and entertainment, which will prompt them to use their mobile phones and other Internet devices more frequently, which will lead to IA in the long run (Parker and Plank, 2015). In addition, PA can reduce the frequency of Internet use by university students by improving their emotional state (Slla et al., 2020; Sheng et al., 2022). From a neurobiological perspective, PA can modulate the concentration of neurotrophic factors and glucocorticoid levels in college students, improve the structure and function of specific regions of the central nervous system, regulate dopamine and its receptors in both directions, and effectively correct college students’ IA behavior (Irene et al., 2020). In addition, appropriate PA stimulates the pituitary gland to secrete endorphins, which bring positive emotional experiences and improve the coping style (CS) of college students, thus alleviating the discomfort of being away from the Internet (Hu and Zhang, 2016; Jenin et al., 2019). From the perspective of personality traits, meta-analysis found that PA was significantly associated with personality traits such as extra-version, dutifulness and neuroticism (Kathryn, 2015). Active participation in PA helps university students to develop stable personality traits and to face the effects of perceived stress with proper CS. In addition, PA can reduce problems in peer interaction and difficult behaviors (Yan et al., 2022), improve interpersonal relationships and life satisfaction, and increase self-awareness and self-efficacy among IA students (Guan et al., 2019).

CSs are a means for individuals to deal with stressful situations and maintain psychological balance during stress (Sheng et al., 2018a). Sawyer et al. (2009) found that negative CS increased the likelihood of depression. Liu et al. (2019) found that CS in adolescents had a greater effect on the onset of anxiety. Thus, CS may be a key variable influencing IA, with positive coping improving IA; poor coping triggering IA. Previous studies have mostly explored the effects of PA on IA, and few studies have explored the mediating role of CS in the process of PA influencing IA.

**Figure 1:**
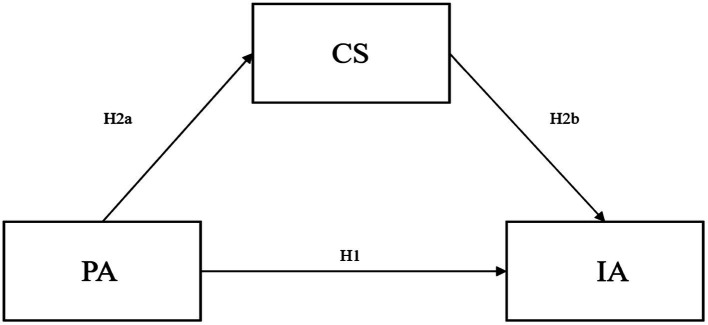
Hypothetical model diagram.

Based on the above discussion, the following hypothesis is proposed in this paper (the hypothetical model is shown in Figure 1):

*H*1: PA is negatively correlated with IA among university students.

*H*2: CS plays a mediating role in the influence of PA on IA.

*H*2a: PA was positively correlated with CS among university students.

*H*2b: CS is negatively correlated with IA in university students.

## Materials and methods

### Participants

The study was conducted in strict accordance with the Declaration of Helsinki and was approved by the Ethics Review Committee of Huabei Normal University for the anonymous survey procedures involved. Informed consent was provided to all participants by members of the research team prior to the formal commencement of the survey. Students were informed of: the purpose of the study, its significance and the use of the data.

In March 2022, we randomly selected 80 “post-00s” university students from Huaibei Normal University to conduct a pre-survey by means of an on-site questionnaire. The pre-survey process was strictly anonymous and confidential, with the same person reading out the instructions and explaining how to answer the questions and the precautions to be taken throughout the test. The questionnaires were collected promptly after completion. The collected data were used to evaluate the reliability and validity of the questionnaire. The official questionnaire was distributed from April to July 2022, using a convenience sampling method, and was administered to “post-00s” university students at five universities in Anhui Province through the Questionnaire Star online platform, where the learning environment was restricted to the school. One hundred questionnaires were distributed to each university. Ten subjects were covered: literature, engineering, science and art. The questionnaire was introduced with basic precautions, “login verification” and “one answer only,” to ensure that respondents did not repeat the question and that no duplicate data would appear.

In terms of questionnaire quality control, we screened the data quality of the questionnaire based on the external and internal characteristics of the questionnaire. Referring to Liu (2015) study, proper questionnaire completion time is a basic guarantee of questionnaire completion quality. Therefore, we took the questionnaire completion time as an important external characteristic to measure the data quality of the questionnaire and eliminated those questionnaires with too short response time. In terms of the internal characteristics of the questionnaire, we identify the logical relationships between the different questions within the questionnaire, using a combination of data detection and automatic jumping functions provided by the web questionnaire system. For mutually exclusive questions in the web questionnaire, unless a neutral answer is chosen (e.g., “generally”), the same participant’s answers to mutually exclusive questions should also be mutually exclusive. If the answers of the same participant do not conform significantly to the internal characteristics of a web-based questionnaire, then it is likely that the questionnaire was not read and completed carefully by the respondent and is therefore defined as a low quality questionnaire.

For the purposes of the study, we used the G-Power to estimate the sample size needed for this study. According to Francesco et al. (2014), the lower limit for the sample size of the pre-test is 3–5 times the number of items in the most extensive sub-scale. A total of 500 questionnaires were distributed, and after excluding invalid questionnaires with missing answers, wrong answers, and obvious discrepancies in feedback, 410 questionnaires were valid, with a validity rate of 82%, which basically met the above requirements.

### Measures

#### Chinese internet addiction scale

The scale was developed by Chen et al. (2003), consists of 26 questions using a Likert 4-point scale and contains five dimensions, namely tolerance (α = 0.863), compulsive (α = 0.876), withdrawal symptoms (α = 0.881), interpersonal health problems (α = 0.874) and time management problems (α = 0.885). The higher the total score, the higher the trend of IA. According to the CIAS-R diagnostic criteria. If the total score was ≥58, the initial screening was for potential IA. If the total score was ≥68, the person was diagnosed as an Internet addict. The scale has high reliability and validity, with a retest reliability of 0.86. The CFA model showed a satisfactory fit (*χ*^2^/*df* = 4.328, GFI = 0.926,AGFI = 0.937, RMSEA = 0.031, CFI = 0.951, NFI = 0.963, RFI = 0.957, SRMR = 0.033).

#### Coping styles scale for Chinese university students

The scale was developed by Liu (2009) and consists of 64 questions using a Likert 5-point scale, ranging from “1-basically all use this approach” to “5-no use this approach.” It consists of 3 dimensions: the problem orientation pathway (α = 0.87), the emotional orientation pathway (α = 0.83) and the avoidance pathway (α = 0.86). For a given dimension, a high score indicates less use of this CS and a low score indicates more use of this style. The scale has high reliability and validity, with a retest reliability of 0.84. The CFA model shows a satisfactory fit (*χ*^2^/*df* = 3.915, GFI = 0.981, AGFI = 0.943, RMSEA = 0.03, CFI = 0.912, NFI = 0.943, RFI = 0.946, SRMR = 0.023).

#### International physical activity scale short form (IPAQ)

The scale was revised by Qu and Li (2004) and consists of seven questions. Except for the last question which investigates the subject’s sedentary status, the first 6 questions investigate the individual’s PA. The IPAQ has been widely used in studies related to PA and has high reliability and validity (Li and Zhang, 2015). Referring to Zhang et al. (2020) study, subjects were divided into 3 groups of high, medium and low exercise according to the grouping criteria in Table 1. During data processing, the energy expenditure value (referred to as MET) of each university student PA was calculated by strictly following this scale to deal with outliers as well as the weights of different intensity physical activities. The MET was used to reflect the PA of university students, with the higher the value taken, the higher the level of PA. In order to make the MET variable more normally distributed, its natural logarithm is taken to construct a continuous variable that conforms to a normal distribution. In this study, the scale has high reliability and validity, with a retest reliability of 0.87. The CFA model showed satisfactory fit results (*χ*^2^/*df* = 4.136, GFI = 0.932, AGFI = 0.951, RMSEA = 0.05, CFI = 0.981, NFI = 0.954, RFI = 0.976, SRMR = 0.033).

**Table 1:** Criteria for grouping individual physical activity levels.

Grouping	Standards
High exercise group	Satisfy any of the following:
	1. Three days or more of all types of high intensity physical activity with a total weekly physical activity level greater than or equal to 1,500 MET-min/w
	2. Three levels of physical activity at a combined intensity of >7 days and a total weekly physical activity level of ≤3,000 MET-min/w
Intermediate exercise group	Satisfy any of the following:
	1. 20 min or more of all types of vigorous physical activity per day for a total of 3 days or more
	2. 30 min or more of all types of moderate intensity and/or walking activity per day for a total of 5 days or more
	3. Three physical activity of 3 intensities for a total of 5 days or more with a weekly total physical activity level greater than 600 MET-min/w
Low exercise group	Failure to report any exercise, or level of exercise that does not meet the criteria for the medium and high groups

### Statistical methods

Data processing and analysis were performed using SPSS 25.0 and Amos 24.0 software. Firstly, a one-way ANOVA was conducted for PA, CS and IA using SPSS 25.0; Pearson’s r correlation coefficient was used to analyse the correlation between the dimensions of PA, CS and IA. Secondly, structural equation modeling was conducted using Amos 24.0 software and a mediating effect model was fitted. Finally, mediating effects were calculated using a macro procedure in SPSS 25.0 with a Bootstrap repeated 5,000 times sample correction with 95% confidence intervals, where the 95% confidence interval did not contain 0. The significance level for this study was set at *p* < 0.05.

## Results

### Common method bias test

As this study used questionnaires to collect research data, there may be common method biases, which may affect the statistical results. Therefore, the results of this study were tested using the one-way test. The results showed that there were six factors with eigenvalues greater than one, and the percentage of variance explained by the first common factor was 31.48%, which was less than the critical threshold of 40%, so it was concluded that there was no serious common method bias in this study.

### Descriptive statistical analysis

In the sample selected for this study, the majority of university students had moderate to high PA and possessed good exercise habits. Out of 410 questionnaires, 47 met the criteria for IA, with a detection rate of 11.46% for IA. There was a statistically significant difference in PA between college students of different genders (*p* < 0.01), with male students having a higher amount of PA; there was no significant difference in CS and IA between college students of different genders (*p* > 0.05), as shown in Table 2.

**Table 2:** Descriptive statistical analysis of age, PA, CS and IA.

Variable	Gender	*M ± SD*	*t*	*p*
Age	M	19.93 ± 1.57	2.568	0.782
	F	19.22 ± 1.09	2.391	0.994
PA	M	1990.06 ± 134.89	10.834	0.001
	F	1357.03 ± 111.42	9.236	0.004
IA	M	55.47 ± 13.28	4.534	0.431
	F	55.31 ± 9.96	5.657	0.767
CS	M	60.17 ± 10.80	4.281	0.363
	F	60.38 ± 9.27	3.546	0.859

### Differential analysis of university students’ PA on CS and IA

The subjects were divided into 3 groups according to the amount of PA, high, medium and low, and the effects of different amounts of PA on CS and IA were analyzed. The results showed that there was a statistically significant difference (*p* < 0.01) between the CS and IA of university students in the different exercise participation groups. The test results showed that the CS scores of the high exercise participation group were significantly higher than those of the other two groups. At the same time, the IA scores in the high exercise participation group were also significantly lower than those in the low and moderate exercise participation groups (see Table 3).

**Table 3:** One-way ANOVA for the effect of different amounts of PA on CS and IA.

Variable	Low exercise participation group (*n* = 113)	Medium exercise participation group (*n* = 163)	High exercise participation group (*n* = 134)	*F*	*p*
CS	56.64 ± 10.17	60.10 ± 8.93	63.47 ± 10.04	15.34	<0.01
IA	59.23 ± 13.78	55.58 ± 10.87	51.96 ± 11.18	11.78	<0.01

PA, CS and IA were all statistically significantly correlated with each other, which provided the basis for the mediating effect test. A negative correlation was found between CS and IA (*r* = −0.560), indicating that the stronger the CS, the lower the degree of IA. A negative correlation was also found between PA and IA (*r* = −0.236), indicating that the greater the amount of PA, the lower the degree of IA. A positive correlation was found between the amount of PA and CS (*r* = 0.278), indicating that an increase in the amount of PA promotes an increase in CS as shown in Table 4. Thus, PA not only influences CS in university students, but also has a positive effect on IA, i.e., as the amount of PA increases, the stronger CS follows and the degree of IA decreases.

**Table 4:** Results of the correlation analysis of PA volume, CS and IA for university students.

Variable	PA	CS	IA
PA	1.000		
CS	0.278**	1.000	
IA	−0.236**	−0.560**	1.000

**p < 0.01.

### Test for mediating effects of CS

To explore whether CS plays a mediating role in the effect of PA on IA among university students, the magnitude of the mediating effect was explored with PA as the independent variable, IA as the dependent variable and CS as the mediating variable. A mediating effects regression analysis of CS was conducted according to the mediating effects test procedure proposed by Wen and Ye (2014) (see Table 5). PA negatively predicted IA (*B* = −0.198, *p* < 0.01). The negative predictive effect of PA on IA remained significant after the inclusion of mediating variables (*B* = −0.119, *p* < 0.01). In addition, PA positively predicted CS (*B* = 0.986, *p* < 0.01) and CS negatively predicted IA (*B* = −0.065, *p* < 0.01).

**Table 5:** Regression analysis results.

Dependent variable	Independent variable	*B*	*t*	*R* ^2^	*F*
IA	Sex	0.083	2.105*	0.052	4.217**
	PA	−0.198	−4.311**		
CS	Sex	0.052	1.086	0.003	5.427*
	PA	0.986	1.121**		
IA	Sex	0.145	2.754	0.287	214.669**
	PA	−0.119	−2.741**		
	CS	−0.065	−7.337**		

B, standardized coefficients; *R*^2^, R square change; F, F change; *p < 0.05; **p < 0.01.

**Figure 2:**
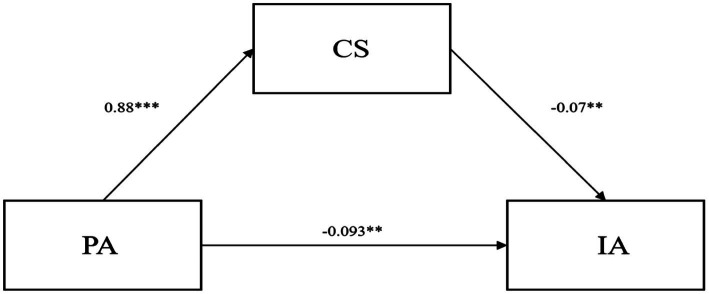
Diagram of the CS mediation model. **p < 0.01; ***p < 0.001.

As can be seen from Table 6, the direct path effect value for PA → IA is-0.093. 95% confidence interval is [−0.139, −0.038], which does not contain 0. This indicates that the path effect is significant, i.e., PA can directly affect IA. While the indirect path effect value for PA → CS → IA is-0.087. 95% confidence interval is [−0.103, −0.057], which does not contain 0. contain 0. This indicates that the pathway effect is also significant. This suggests that PA not only improves IA among college students directly, but also reduces IA by enhancing CS. CS partially mediates the effect between PA and IA among college students, with the mediating effect accounting for −0.087/[(−0.093) + (−0.087)] = 48.33% of the total effect, and the mediating model for CS is shown in Figure 2.

**Table 6:** Bootstrap analysis of the mediating effect test.

Paths	Effect	Boot SE	Bias-corrected 95% CI		Effect size ratio
			Lower limit	Upper limit	
PA → IA	−0.093	0.029	−0.139	−0.038	51.67%
PA → CS → IA	−0.087	0.012	−0.103	−0.057	48.33%
Total effect	−0.180	0.033	−0.215	−0.106	100%

## Discussion

### The impact of PA on university students’ IA

The results of this study showed that college students were moderately physically active overall. This result is consistent with previous studies (Chen and Zhang, 2021). A study by Dong and Mao (2021) found that a significant number of university students were occupied with Internet use and sedentary time during their leisure time, and they were reluctant to go for PA, leading to a decrease in the amount of PA (Harvey and Chastin, 2013). Another study found that low-intensity PA, such as slow walking and strolling, has become the main form of PA performed daily by most university students (Zhang, 2018). Therefore, college students’ PA levels are at an intermediate level.

The results of this study showed that there was a statistically significant difference between the PA levels of male and female university students, in terms of gender differences. This result is consistent with previous studies (Li et al., 2018). The reason for this is that most female university students prefer low-intensity, non-confrontational PA, such as walking, jogging, yoga and aerobics. Male university students prefer moderate-to-high intensity, competitive PA, such as ball games and HIIT (Zhang and Lei, 2015). In addition, gender role conflict is considered to be an important factor influencing female university students’ participation in PA. Compared to male university students, female university students are more likely to develop negative emotions such as anxiety and distress. Male university students showed significantly higher levels of interpersonal support and exercise identity when participating in exercise than female university students (Li et al., 2015; Zhang and Lei, 2015). Therefore, male college students had relatively higher levels of PA than female college students.

The results of this study showed that the IA detection rate was about 11.46%, a result similar to that of previous studies. However, differences in sample size, differences in scale selection and judgment criteria, or differences in testing time and region can affect the detection rate of addiction. Some studies have shown that the prevalence of IA is higher among boys than girls (Zhang and Lei, 2015), especially in the category of online gaming addiction (Cuhadar, 2012). Some studies have also found the opposite, showing that the relationship between gender and IA is not significant (Bi, 2016). However, overall, male students’ interest in online games and money management and female students’ preference for shopping and dating apps are significant factors in the increase of IA (Long et al., 2016). Other studies have found that college freshmen have the lowest rates of IA, and that the rate of IA among college students increases with grade level (Brand et al., 2014). As “post-00” college students enter school independently, the unfamiliar environment can easily bring about emotional and psychological problems, prompting them to turn to other alternatives for inner satisfaction. As a result, university freshmen are at a high risk of IA, and the proportion of IA shows a gradual increase as the year progresses. In addition, the increasing convenience and cheapness of Internet access for university students, influenced by a combination of factors such as the upgrading of smartphones, the popularity of online games and decreasing Internet tariffs, are also important factors in the increase of IA.

The results of this study showed a significant negative effect of PA on IA. This result is consistent with previous studies (Qin et al., 2020; Guo et al., 2022) and also verifies hypothesis *H*1. The higher the level of PA, the less likely IA will occur. This suggests that PA, as an economic and effective measure to improve IA, has a preventive and curative effect on IA. PA and the Internet serve as an important part of university students’ life and entertainment. The difference between the two is that the internet provides a virtual world for university students, while PA is a means of entertainment and health promotion for university students in the real world. Exercise intervention therapy argues that PA can crowd out time spent using the Internet and to some extent replace the gaming and dating functions of the Internet. Currently, fitness apps are being introduced to promote PA among university students to a certain extent. Therefore, PA is not only effective in improving IA among university students, but also helps to promote physical and psychological health, increased willpower and more self-discipline (Ma and Huang, 2020).

### The mediating effect of CS between PA and IA

The results of the mediating effect in this study show that, on the one hand, PA can directly influence IA, indicating that college students can divert their attention, squeeze time for Internet use and satisfy their psychological needs through PA, thus reducing IA. On the other hand, PA can also indirectly influence college students’ IA through CS, which plays a mediating role between PA and college students’ IA, and the proportion of the mediating effect to the total effect is 48.33% The hypothesis *H*2 was verified. Specifically, PA significantly and positively predicted CS, which is consistent with previous studies (Fedewa and Ahn, 2011; Sheng et al., 2018b), i.e., confirming the association between PA and CS in university students, and hypothesis *H*2a was verified. Fedewa and Ahn (2011) argued that long-term PA could promote changes in cognitive function and thus coping ability in college students, and Sheng et al. (2018a,b) showed that PA can directly influence individuals’ CSs and improve their self-confidence; Wang and Gao (2018) concluded that fitness qigong can improve individuals’ CSs. The above research results support the positive effect of PA on CS. However, differences in the effects of different exercise programs on CS intervention can be studied comparatively in the future.

The results of the present study showed that CS significantly and negatively predicted IA, which is consistent with previous research (Wei et al., 2022), i.e., confirming the association between CS and IA in university students, while hypothesis *H*2b was tested. In the field of clinical psychology, IA is considered an impulse control disorder. IA is an addiction to coping behavior and is a form of negative coping. The results of the study showed that Internet-addicted college students scored low on CS choices such as problem solving and help seeking, and high on self-blame and avoidance. This suggests that Internet-addicted college students tend to adopt relatively negative CSs in their coping strategies. This result is similar to previous research (Slla et al., 2020). For Internet-addicted students, the virtual world created by the Internet is used as a tool for emotional catharsis and escape from reality. The internet is used to vent negative emotions and to escape from the difficulties and frustrations they face in real life, thus maintaining psychological balance. However, negative CSs can not only bring about a variety of psychological problems, but also affect the physical health of university students. On the contrary, if students are supported and guided to develop positive ways of coping, it will be conducive to the healthy physical and mental development of Internet addicted students and will have positive implications for the prevention and cessation of IA.

The results of this study show that CS can play a mediating role between PA and IA in university students, further enriching the previous research findings. The mediating effect of CSs lies in the fact that the improvement of individuals’ physiological indicators through a period of PA will promote individuals to choose more positive CSs, thus achieving prevention and improvement of IA. This result can be explained by the psychological stress theory as well as the theory of internet use and satisfaction. The psychological stress theory (Zhang et al., 2021) suggests that when individuals are exposed to stressful situations, two different psychological responses are generated, with positive psychological responses facilitating the production of correct cognitive evaluations and negative psychological responses preventing normal coping abilities from being developed. Internet use and satisfaction theory further states that individuals using the Internet can satisfy their psychological needs and promote positive attitudes toward the Internet, which can lead to IA in the long run. Some researchers argue that Internet-addicted college students often tend to adopt a single standard of evaluation to assess their abilities and values, which leads to biased conclusions and even to enter the virtual world of the Internet to seek psychological comfort (Chu et al., 2016). Some researchers have also suggested that individuals with higher scores on the IA Measure may use the services and functions of the Internet as a tool to relieve stress and accordingly engage in less PA (Zhang et al., 2022).

Through the above discussion, we found that PA can not only influence college students’ IA directly, but also indirectly through CS as a mediating pathway. Therefore, in the daily life of college students, schools should develop more abundant and effective forms of physical activities to encourage and guide college students to PA. This will help to alleviate the discomfort and negative emotions that arise when students are away from the Internet and to prevent and improve IA among students.

### Limitations

It is important to note the limitations of this study. This study is a cross-sectional study, which may have implications for revealing the causal relationships between variables. In future studies, longitudinal studies could be used to reveal the mechanism of the effect of PA on IA. An experimental approach could also be used to better control for the effects of other variables. In addition, potential mediating and moderating variables between PA and IA could be further explored. In future studies, the scope of investigation can be expanded to make a more prominent theoretical contribution to the study of the effects of PA on IA.

## Conclusion

A high level of moderate PA is more likely to reduce IA symptoms and problems across all dimensions in college students. A high level of moderate PA was more likely to increase CS in college students, and more positive CS was more likely to reduce IA symptoms in college students. CS partially mediated the relationship between PA and IA. These findings may provide insight into the prevention and treatment of IA among Chinese university students.

## Data availability statement

The original contributions presented in the study are included in the article/supplementary materials, further inquiries can be directed to the corresponding authors.

## Ethics statement

The studies involving human participants were reviewed and approved by Huaibei Normal University. The patients/participants provided their written informed consent to participate in this study.

## Author contributions

M-cC designed the study and wrote the manuscript. YW and RZ collected and analyzed the data. M-cC and SW revised the manuscript. L-kQ provided significant revision input and fund support. All authors contributed to the article and approved the submitted version.

## Funding

This study was supported by the Youth Fund for Humanities and Social Sciences Research of the Ministry of Education (Project No. 19YJC890045), 2021 Huaibei Normal University-Yongcheng Jiang Han Sports Development Company Limited Horizontal Research Project (Project No. 22200121), 2021 Anhui Provincial Teaching Quality Project (Project No. 2021jyxm1330), and 2020 Huaibei Normal University School-level Teaching and Research Project (Project No. 2020xjxyj027).

## Conflict of interest

The authors declare that this study received funding from 2021 Huaibei Normal University-Yongcheng Jiang Han Sports Development Company Limited Horizontal Research Project (Project No.22200121). The funder had the following involvement in the study: Data collection and analysis.

## Publisher’s note

All claims expressed in this article are solely those of the authors and do not necessarily represent those of their affiliated organizations, or those of the publisher, the editors and the reviewers. Any product that may be evaluated in this article, or claim that may be made by its manufacturer, is not guaranteed or endorsed by the publisher.
